# Recovery of Nontuberculous Mycobacteria and Nocardiae Causing Skin/Soft Tissue Infections by Use of the Copan ESwab Collection and Transport System

**DOI:** 10.1128/JCM.01302-19

**Published:** 2019-12-23

**Authors:** B. Gandhi, G. Woods, T. Mazzulli

**Affiliations:** a Department of Microbiology, Mt. Sinai Hospital, Toronto, Ontario, Canada; b Department of Laboratory Medicine and Pathobiology, University of Toronto, Toronto, Ontario, Canada; c Department of Pathology, University of Arkansas, Little Rock, Arkansas, USA; Cepheid

**Keywords:** Copan ESwab, nontuberculous mycobacteria, viability

## LETTER

Recent reports have shown that infections with nontuberculous mycobacteria (NTM) are increasing worldwide and often are difficult to diagnose and to treat (1–3). Consequently, improved diagnostics are needed. Clinical and Laboratory Standards Institute (CLSI) guidelines (4) describe methods for assessing the ability of transport devices to maintain microorganisms in a viable condition for up to 48 h during transport at room temperature (RT) (20 to 25°C) and refrigerator temperature (FT) (2 to 8°C). There are no formal published data on the viability of NTM using swab-based systems; however, a study conducted only at RT was presented at the 110th General Meeting of the American Society for Microbiology (5). The present investigation was designed, using CLSI methods, for evaluation of the Copan ESwab transport system (Copan Diagnostics Inc., Murrieta, CA) for NTM species commonly causing skin and soft tissue infections.

Nine NTM clinical isolates (Mycobacterium porcinum, Mycobacterium abscessus subsp. *abscessus*, Mycobacterium senegalense, Mycobacterium mucogenicum, Mycobacterium fortuitum, Mycobacterium avium, Mycobacterium chelonae, Mycobacterium marinum, and Mycobacterium haemophilum), which had been previously identified using the Bruker MALDI Biotyper CA system (Bruker Daltonics Inc., Billerica, MA, USA), and Nocardia asteroides ATCC 19247 were evaluated in this study. After 5 to 7 days of growth on blood agar plates, a heavy stock suspension of each organism was prepared in 4.0 ml of sterile saline and vigorously vortex mixed. From this, suspensions were prepared in triplicate and adjusted to match a 0.5 MacFarland turbidity standard (1.5 × 10^8^ CFU/ml), using a Vitek nephelometer (6). Each suspension was serially diluted 1:10 in normal saline to obtain working suspensions of ∼1.5 × 10^7^ CFU/ml to ∼1.5 × 10^3^ CFU/ml. One hundred microliters of each working suspension was placed in 18 wells of a microtiter plate. Flocked swabs were placed in the wells, allowed to absorb the inoculum for ∼10 s, and returned to their respective labeled ESwab transport vials. Each microorganism/device combination was assayed in triplicate at three time points (0, 24, and 48 h). The 0-h swabs were removed from the transport vials, containing 1.0 ml of Amies broth, after ∼15 min of incubation and discarded; 100 μl of the inoculum was plated onto a Middlebrook 7H10 agar plate, streaked for isolation, and incubated at 30°C in O_2_. The remaining ESwabs were incubated at RT or FT for 24 or 48 h. Results were calculated by taking an average of the colony counts from triplicate tests. Counts at 24 and 48 h were compared to 0-h reference counts.

Results are summarized in Table 1. The CLSI criterion for acceptable recovery using the roll plate method (i.e., ≥5 colonies recovered from the dilution yielding a baseline count closest to 250 colonies [1]) was met for 6 of the 10 organisms tested at 24 h in RT incubation. Of these 6, 5 also met the CLSI criterion at 48 h at RT. Another 4 NTM organisms met the criteria within 10% at 48 h at RT. Nine organisms produced CFU within ∼50% of the baseline CFU at 24 h and 48 h at FT. The exception was M. marinum, which showed overgrowth after 48 h of storage. At the 10^6^ dilution, 7 organisms yielded colonies too numerous to count (TNTC). At 10^5^ and/or 10^4^ dilutions, the same 7 yielded countable colonies within ∼250 CFU/ml at both 24 and 48 h, compared to 0-h counts, except for M. marinum, which at 10^5^ yielded colonies TNTC at 48 h at RT. M. mucogenicum, M. fortuitum, and N. asteroides had lower counts and produced countable colonies at 10^6^ dilution. Mycobacterial cells are a bit larger than routine bacteria; therefore, initial serial dilutions made from a 0.5 McFarland standard contained the desired concentrations of bacteria at higher dilutions.

**TABLE 1:** Recovery of NTM and *N. asteroides* held for 24 or 48 h at RT or FT, using the Copan ESwab transport system

Organism and starting concn (CFU/ml)^*a*^	Recovery (CFU/ml)^*b*^ after:				
	0 h	24 h at RT	48 h at RT	24 h at FT	48 h at FT
M. porcinum					
10^6^	TNTC	TNTC	TNTC	TNTC	TNTC
10^5^	82	75	80	61	48
10^4^	6	4	5	7	5
M. abscessus					
10^6^	TNTC	TNTC	TNTC	TNTC	TNTC
10^5^	335	370	276	293	284
10^4^	36	43	27	35	30
M. senegalense					
10^6^	TNTC	TNTC	TNTC	TNTC	TNTC
10^5^	246	241	237	238	239
10^4^	30	30	32	25	25
M. mucogenicum					
10^6^	107	108	108	112	106
10^5^	4	6	5	5	6
10^4^	0	0	0	0	0
M. fortuitum					
10^6^	205	290	240	184	155
10^5^	12	17	14	12	12
10^4^	1	3	2	1	1
M. avium					
10^6^	TNTC	TNTC	TNTC	TNTC	TNTC
10^5^	143	150	152	129	123
10^4^	17	19	21	16	17
M. chelonae					
10^6^	TNTC	TNTC	TNTC	TNTC	TNTC
10^5^	270	263	243	242	230
10^4^	25	22	16	17	20
M. marinum					
10^6^	TNTC	TNTC	TNTC	TNTC	TNTC
10^5^	166	235	TNTC	148	138
10^4^	9	22	433	7	6
M. haemophilum					
10^6^	TNTC	TNTC	TNTC	TNTC	TNTC
10^5^	257	282	298	258	257
10^4^	30	33	34	35	28
N. asteroides					
10^6^	125	114	92	91	60
10^5^	11	8	7	11	6
10^4^	0	0	0	0	0

a Tenfold (10^6^, 10^5^, and 10^4^) serial dilutions starting from a 0.5 McFarland standard were made.

b Data are expressed as the average CFU (performed in triplicate).

Our results indicate that the Copan ESwab collection and transport device appears to be an appropriate system for the maintenance, transport, and recovery of select NTM and *Nocardia* species, by maintaining the viability of NTM species known to cause skin and soft tissue infections and an ATCC strain of N. asteroides with fair efficiency for up to 48 h at RT. However, until further studies are carried out in clinical settings to confirm the utility of swab specimens, tissue/fluid samples remain the preferred specimen type for detection of NTM. Studies directly comparing flocked swabs to paired tissue/fluid specimens are needed. Further study is also required to determine the utility of the ESwab in recovering NTM and *Nocardia* species from swab specimens collected from patients with suspected infections.

## References

[1] Atkins BL, Gottlieb T 2014 Skin and soft tissue infections caused by nontuberculous mycobacteria. Curr Opin Infect Dis 27:137–145. doi:10.1097/QCO.0000000000000041. 24464139

[2] Gonzalez-Santiago TM, Drage LA 2015 Nontuberculous mycobacteria: skin and soft tissue infections. Dermatol Clin 33:563–577. doi:10.1016/j.det.2015.03.017. 26143432

[3] Misch EA, Saddler C, Davis JM 2018 Skin and soft tissue infections due to nontuberculous mycobacteria. Curr Infect Dis Rep 20:6. doi:10.1007/s11908-018-0611-3. 29556857

[4] Clinical and Laboratory Standards Institute. 2010 Quality control of microbiological transport systems; approved standard—2nd ed CLSI document M40-A2 Clinical and Laboratory Standards Institute, Wayne, PA.

[5] Snyder JW, Munier GK, Schiavi CM, Johnson CL 2010 Evaluation of the Copan liquid Amies elution swab (ESwab) for maintaining the viability of selected fungi and mycobacterium spp., abstr F-2141 Abstr 110th Gen Meet Am Soc Microbiol.

[6] Preece CL, Perry A, Gray B, Kenna DT, Jones AL, Cummings SP, Robb A, Thomas MF, Brodlie M, O'Brien CJ, Bourke SJ, Perry JD 2016 A novel culture medium for isolation of rapidly-growing mycobacteria from the sputum of patients with cystic fibrosis. J Cyst Fibros 15:186–191. doi:10.1016/j.jcf.2015.05.002. 26002312

